# Role of condom negotiation on condom use among women of reproductive age in three districts in Tanzania

**DOI:** 10.1186/1471-2458-12-1097

**Published:** 2012-12-20

**Authors:** Amon Exavery, Almamy M Kanté, Elizabeth Jackson, John Noronha, Gloria Sikustahili, Kassimu Tani, Hildegalda P Mushi, Colin Baynes, Kate Ramsey, Ahmed Hingora, James F Phillips

**Affiliations:** 1 Ifakara Health Institute (IHI), Dar es Salaam, Tanzania; 2 Heilbrunn Department of Population and Family Health, Mailman School of Public Health, Columbia University, Columbia, USA

**Keywords:** Condom negotiation, Condom use, Women, Rural Tanzania

## Abstract

**Background:**

HIV/AIDS remains being a disease of great public health concern worldwide. In regions such as sub-Saharan Africa (SSA) where women are disproportionately infected with HIV, women are reportedly less likely capable of negotiating condom use. However, while knowledge of condom use for HIV prevention is extensive among men and women in many countries including Tanzania, evidence is limited about the role of condom negotiation on condom use among women in rural Tanzania.

**Methods:**

Data originate from a cross-sectional survey of random households conducted in 2011 in Rufiji, Kilombero and Ulanga districts in Tanzania. The survey assessed health-seeking behaviour among women and children using a structured interviewer-administered questionnaire. A total of 2,614 women who were sexually experienced and aged 15-49 years were extracted from the main database for the current analysis. Linkage between condom negotiation and condom use at the last sexual intercourse was assessed using multivariate logistic regression.

**Results:**

Prevalence of condom use at the last sexual intercourse was 22.2% overall, ranging from12.2% among married women to 54.9% among unmarried (single) women. Majority of the women (73.4%) reported being confident to negotiate condom use, and these women were significantly more likely than those who were not confident to have used a condom at the last sexual intercourse (OR = 3.13, 95% CI 2.22-4.41). This effect was controlled for marital status, age, education, religion, number of sexual partners, household wealth and knowledge of HIV prevention by condom use.

**Conclusion:**

Confidence to negotiate condom use is a significant predictor of actual condom use among women in rural Tanzania. Women, especially unmarried ones, those in multiple partnerships or anyone needing protection should be empowered with condom negotiation skills for increased use of condoms in order to enhance their sexual and reproductive health outcomes.

## Background

Confidence to negotiate safer sex practices is very crucial especially today when the Acquired Immune Deficiency Syndrome (AIDS) due to Human Immunodeficiency Virus (HIV) is rampant. In the global warfare against HIV/AIDS, research underscores the importance of communication between sexual partners concerning condoms use [1,2]. This is based on the fact that communication between sexual partners about condom use is associated with increased use of condoms [3]. More importantly and probably beyond communication, it is established that those who convince or persuade their sexual partners to use condoms are more likely to actually use them than those who do not [4].

A condom - if used correctly and consistently - guarantee more than 90% effectiveness at preventing heterosexual acquisition and transmission of HIV [5,6]. Family planners also acknowledge condoms as players of an imperative role in reducing the risk of unintended pregnancies, with their effectiveness estimated at 85-98% and 79-95% for male and female condoms respectively [7]. Therefore, promotion of condom use has been and continues to receive considerable attention in fighting the HIV/AIDS pandemic [8], and this is very important particularly in sub-Saharan Africa (SSA) where unprotected heterosexual contact involving an infected partner is a major pathway for HIV transmission [9-12].

Although evidence showing an increased use of condoms over the past decade exists [8,13], negative attitudes towards condom use reign mainly due to factors such as fertility desires and sexual conformity of women as a way to accomplish their economic status [14]. Furthermore, barriers to condom use incline towards cultural definition of a good sex and perceptions of sex from a procreation standpoint [15,16]. In addition, research shows that rejection of condom use is due to several reasons including assertions that it reduces sex enjoyment [16], uncomfortable to use, they come off inside a woman [17,18] and that they pedal promiscuity [15]. Other barriers such as doubt in the efficacy of condoms, myths, physical side-effects and others have also been reported [19]. On the other hand, marital status greatly affects condom use. In non-marital relationships, condom use is high and chunkily intended for preventing sexually transmitted infections (STI) especially HIV/AIDS. In contrast, condom use and marital intimacy are incompatible, since bringing the two together may be thought of as confessing infidelity [20]. Evidence shows that other than preventing a pregnancy, condom use within marriage suggests lack of trust between partners and consequently betrays the intimacy that is necessary within a marital relationship [20,21]. Married women will most likely use condoms if they know or suspect that their partners are infected with HIV or other STIs. Condom use among unmarried women may be affected by the type of partner. Relationships in which sugar daddies or large amounts of material assistance are involved, condom use is less likely [22]. Also, condom use tends to be higher in the beginning of a relationship, but drops in subsequent contacts as the relationship extends, even if the HIV status among the partners may be unknown [17].

Gender inequality in the HIV/AIDS burden has been reported in SSA [23,24], thus a need for gender-specific efforts in combating the HIV/AIDS. Evidence shows that in 2007, women accounted for 61% of all adults living with HIV in SSA, and 75% of young people infected were girls [13]. The extent of HIV infection tends to be higher among women than men. It has been established that the biological make-up of the female genitalia together with cultural frameworks within which sex occurs, exposes women more to the risk of contracting HIV than their male counterparts [25]. Similarly, while the overall HIV prevalence in Tanzania in 2008 was 6% among adults, so was 7% among women and 5% among men [26].

There is a widespread knowledge of HIV/AIDS prevention methods among both women and men in Tanzania. Recent statistics from the Tanzania Demographic and Health Survey (TDHS) reveal that the proportion of women and men who knew that the chance of becoming infected with the HIV is reduced by limiting sexual intercourse to one uninfected partner who has no other sexual partners was 87% and 90% respectively. Likewise, 76% of both men and women knew that the chance of contracting HIV/AIDS is reduced by using condoms. Over 70% of men, and women reported being knowledgeable of both methods [27]. However, HIV/AIDS knowledge about transmission and prevention is not enough if people especially those needing protection feel powerless to negotiate safer sex methods with their sexual partners and consequently use them during sexual contacts. Evidence shows that condom use at the last premarital sex among women in Tanzania increase with education, and being in the age group 15-19 than 20-24 [26]. Another recent study on condom use among bar maids in Tanzania reveal a significantly low likelihood of condom use among girls who drink alcohol compared to those who do not; and 10-14 year-olds compared to women aged at least 30 years [28]. It is further shown that type of the sex partner counts in the decision-making about condom use. A study which was conducted recently among female students in Dar es Salaam, Tanzania showed that deciding about condom use with a boyfriend was made by both partners or a female partner alone whereas deciding about condom use with a sugar daddy was predominantly made by the sugar daddy himself [22]. However, even though these and other correlates of condom use among women have been identified [29,30], to our knowledge, we are aware of no existing attempt so far that has assessed the relationship between condom negotiation and condom use among women in rural settings of Tanzania. Therefore, this study addresses this gap with the following objectives: (1) to determine the proportion of women with confidence to negotiate condom use with their sexual partners, (2) to describe the prevalence of condom use at the last sexual intercourse, and (3) to assess the linkage between condom negotiation and condom use among 15-49 year-old women in three districts (Rufiji, Kilombero and Ulanga) in Tanzania.

## Methods

### Study area and study population

The main survey in which the current study stems was conducted in Rufiji, Kilombero and Ulanga districts of Tanzania in 2011. Within these districts, we used two existing and ongoing Health and Demographic Surveillance Systems (HDSS) of Rufiji and Ifakara to identify the study population. Participants were resident women of the HDSS aged 15-49 years. In 2011, these HDSS altogether had a population of 374,722 people, 24% of which were women of child-bearing ages. Further details about these HDSS are available elsewhere [31,32].

### Study design and sampling

The Ifakara Health Institute (IHI), in partnership with the Mailman School of Public Health/Columbia University (MSPH/CU) currently implements a 5-year project in Rufiji, Kilombero and Ulanga districts in Tanzania with a focus on accelerating achievement of the millennium development goals (MDGs) 4 and 5. The project is called *Connect*, a name derived from its core functions. The project tests interventions to strengthen the continuum of care from a household to a health facility and determining how this impacts child mortality, particularly newborn mortality. This is happening through an intervention of Community-based Health Agents (CHA). A CHA is a paid health worker who is formally trained, equipped and employed by the health system to provide a package of health services in the community; connect people across the household to health facility continuum and engage in risk identification and management. Details about the project are available at [33]. Before the CHA intervention, a baseline household survey was conducted between May and July 2011 to assess health seeking behaviour and service utilization patterns of women and children less than five years of age. The survey was organized as cross-sectional in design. Field data collection was accomplished using a standard interviewer-administered questionnaire which for the most part had closed-ended questions. The questionnaire was organized in nine modules, namely, background characteristics and household composition, water and sanitation, reproduction, family planning, health during pregnancy and antenatal care, intrapartum care, immediate newborn care, infant and under-five health and HIV/AIDS. Data on the HIV/AIDS module were used in the current analysis to investigate the role of condom negotiation on condom use among women.

The Rufiji and Ifakara HDSS data platforms provided a sampling frame of households from which 2,183 households were successfully visited and yielded 3,127 women who were interviewed for the main survey. The households were sampled randomly using Probability Proportionate to Size (PPS) to ensure a representative sample of each village in the study area. From the 3,127 women interviewed 2,614 (83.6%) were aged 15-49 years and sexually experienced, thus analyzed to answer the current research question.

### Variables, definitions and statistical analyses

A dependent or outcome variable for this study was condom use at the last sexual intercourse among sexually experienced women. A woman was considered to be sexually experienced if she was married or living with a partner as married or ever married (divorced or widowed). To determine if an unmarried (single) woman was sexually experienced, we strictly considered two responses, “*yes*” or “*no*”, to the question: “*Thinking of your most recent time having a sexual intercourse*, *did you or your partner use a condom*?” These responses were also applicable to the women who were currently married or ever married. Women whose responses were “*don*’*t know*” or “*no response*” were very few (0.2%), thus excluded from this analysis because it was difficult to ascertain whether or not they were sexually experienced. Removing these women may have had negligible effect to the overall results since the sample was large enough for such a negligible loss of subjects.

Condom use was considered to have occurred if a woman reported that either herself or her male partner wore it during the last sexual intercourse. Otherwise, no condom use was considered to have occurred. This was thus represented as

$$\text{Condom}\;\text{use}=\{\begin{array}{l} 1\;if\;a\;condom\;was\;used\;at\;the\;last\;sexual\;intercourse\;a\;woman\;had\;\\ 0\;if\;no\;condom\;was\;used\;at\;the\;last\;sexual\;intercourse\;a\;woman\;had \end{array}$$

The main independent or explanatory variable was condom negotiation. This variable - condom negotiation - was measured using a single question, similar to Urada and colleagues [34] in their study of condom negotiation among female sex workers. We are however aware of other studies in which this variable was measured using a series of questions [35]. In this study, condom negotiation was derived from the question: “*For you personally*, *would it be*… (*a*) *very easy*, (*b*) *easy*, (*c*) *difficult*, *or* (*d*) *very difficult*… *to ask your sexual partner to use a condom before having sex*?” The categories of this variable, *a*-*d*, were re-grouped by combining the first two (*a* and *b*) and referred to as “confidence to negotiate condom use” and the last two (*c* and *d*) and referred to as “no confidence to negotiate condom use”. This was dictated by the fact that the two categories - *a* and *d* - had fewer responses compared to *b* and *c*, a situation which would have compromised the efficiency of our statistical tests if each category was treated individually.

Other explanatory variables were included as potential confounders. These were socio-demographic variables namely, age, religion, marital status, education attainment, household wealth status, and district of residence. We also included number of sexual partners a woman had in the past 12 months, and knowledge of whether people can reduce their chances of contracting the HIV by using a condom every time they have sex. A detailed description of these variables is presented in Table 1. Household wealth status was constructed using Principal Component Analysis (PCA) of household asset ownership of a toilet, type of the toilet and source of drinking water. Unfortunately, other household assets such as house roofing material, wall material, floor material etc. that are conventionally included in determining household wealth status using PCA were not available. Finally, all women were grouped in three categories of household wealth status as poor, middle or rich according to their household wealth score.

**Table 1:** Description of variables used

**Role**	**Variable**	**Categories**	**Code**
Dependent or outcome variable	1. Did you or your sexual partner use a condom at the last sexual intercourse?	No	0
		Yes	1
Independent or exposure variable of interest	2. Negotiation of condom use	Not confident	0
		Confident	1
Socio-demographic variables	3. Age group (in years)	<20	0
		20-29	1
		30-39	2
		40-49	3
	4. Marital status	Married or in union	0
		Divorced/Widowed (ever married)	1
		Single	2
	5. Religion	Christian	0
		Muslim	1
		Other (e.g. traditional etc.)	2
	6. Educational attainment	Never been to school	0
		Primary	1
		Secondary and higher	2
	7. District of residence	Kilombero	0
		Rufiji	1
		Ulanga	2
	8. Household wealth status	Rich	0
		Middle	1
		Poor	2
Other variables	9. Can people reduce their chances of contracting the AIDS virus by using a condom every time they have sex?	Yes	0
		No	1
		Don’t know	2
	10. Number of sexual partners a woman has had in the past 12 months	At most one	0
		At least two (multiple partners)	1

Our analysis employed both descriptive and analytical techniques of statistics. Frequency distributions of responses by categories of each variable were calculated and presented. Bivariate analyses of condom use by condom negotiation and the rest of the explanatory variables were also calculated and presented in Table 2. The degree of association between each of these relationships was tested using Pearson’s Chi-Square because all variables involved in the cross-tabulations were categorical. Further analysis was performed in a multivariable fashion using logistic regression to assess how condom negotiation related to condom use at the last sexual intercourse. Explanatory variables were selected for inclusion in the multivariate logistic regression if there was an evidence that each variable improved the overall model [36], except the explanatory variable of interest which automatically qualified for inclusion regardless of the significance status in the bivariate analysis. The model was checked for statistical interactions and adequacy before being declared as final using the Hosmer-Lemeshow goodness-of-fit test [37]. Odds ratios (OR), standard errors (SE), 95% confidence intervals (CI) and P-values were all presented. The whole process of data analysis was carried out using STATA (version 11) statistical software.

### Ethics

The main study from which this paper stems was approved by the Medical Research Coordinating committee (MRCC) of the National Institute for Medical Research (NIMR) in Tanzania. During field data collection, participation in the survey was entirely voluntary and each of the respondents signed an informed consent after which an interview followed. For each participant less than 18 years of age, consent was sought from her respective parent/guardian/husband. After the participant or her guarantor signed or provided a thumb print on the consent form, the interviewer also signed the form to verify that the nature, purpose, potential benefits and possible risks associated with her participation in the research were explained to the participant. Storage of the completed consent forms and the questionnaires were separate to evade the possibility of linking the respondent with her responses. Handling of the data was managed by a few experts and the whole process was generally confidential.

## Results

### Demographic characteristics

Table 2 among other things presents socio-demographic and other characteristics of the study participants. The study was conducted on 2,614 women who were aged 30.3 years on average (SD = 9). Almost three-quarters (74.1%) were married, 7% divorced or widowed and 18.9% were single at the time of the survey. In terms of religious beliefs, nearly a half (50.3%) of the participants reported to be Muslims, 46.4% Christians and 3.3% cultural or traditional belief followers. Education attainment was assessed through enquiries on the highest level of education accomplished. While 20.0% reported having never been to school, 70.0% had primary education and only 10.0% had a secondary or higher education. Concerning place of residence, majority (63.4%) resided in Kilombero district. Other districts, Rufiji and Ulanga, hosted 21.2% and 15.4% of the total participants respectively.

**Table 2:** **Distribution of the study participants, and condom use at the last sexual intercourse among 15**-**49 year**-**old women in rural Tanzania by background and other characteristics**, **2011** (**n** = **2**,**614**)

			**Bivariate analysis**	
**Variable**	**Number of women (n)**	**Percent distribution**	**Percent that used a condom at the last sexual intercourse**	**P - value**
**Negotiation of condom use**	2,563	100.0%	22.3%	
Not confident	683	26.7%	6.9%	
Confident	1880	73.4%	27.9%	<0.001
**Age group (in years)**	2,614	100.0%	22.2%	
<20	377	14.4%	48.0%	<0.001
20-29	881	33.7%	23.8%	
30-39	858	32.8%	15.5%	
40-49	498	19.1%	11.2%	
Mean= 30.3, SD=9.0				
**Marital status**	2,614	100.0%	22.2%	
Currently married or living with a partner as married	1,937	74.1%	12.4%	
Divorced/Widowed	183	7.0%	37.7%	
Single	494	18.9%	54.9%	<0.001
**Religion**	2,614	100.0%	22.2%	
Christian	1,212	46.4%	21.9%	
Muslim	1,315	50.3%	23.4%	
Other (e.g. traditional etc.)	87	3.3%	9.2%	0.022
**Educational attainment**	2,614	100.0%	22.2%	<0.001
Never been to school	522	20.0%	14.4%	
Primary	1,828	69.9%	20.1%	
Secondary and higher	264	10.1%	52.3%	
**District of residence**	2,614	100.0%	22.2%	
Kilombero	1,658	63.4%	22.7%	
Rufiji	553	21.2%	21.5%	
Ulanga	403	15.4%	21.1%	0.721
**Household wealth status**	2,614	100.0%	22.2%	
Rich	788	30.2%	25.6%	
Middle	818	31.3%	24.8%	<0.001
Poor	1,008	38.6%	17.4%	
**Can people reduce their chances of contracting HIV by using a condom every time they have sex?**	2,583	100.0%	22.2%	
Yes	1,978	76.6%	24.1%	
No	444	17.2%	18.5%	
Don’t know	161	6.2%	9.3%	<0.001
**Number of sexual partners a woman has had in the past 12 months**	2,611	100.0%	22.2%	
At most one	2,407	92.2%	20.0%	
At least two (multiple partner)	204	7.8%	47.6%	<0.001

P-values are based on Pearson’s Chi-Square test at 5% significance level.

### Knowledge, confidence and number of sexual partners

A large proportion (76.6%) of the women reported knowing that people can reduce their chances of contracting HIV by using a condom every time they have sex. A few (17.2%), however, did not accept the claim while 6.2% reported that they do not know of it. We also found that 73.4% of the women reported being confident to negotiate condom use with their sexual partners. Concerning the number of sexual partners in the previous 12 months, 92.2% of the women reported having had no more than one partner while the rest had at least two sexual partners (i.e. multiple sexual partners). The highest number of sexual partners reported in the past 12 months was 5 and the average was 1.1 (SD = 0.38).

### Condom use at the last sexual intercourse

Each woman was asked in an adequate privacy to report on whether or not a condom was used during the last sexual intercourse she had. This variable was cross-tabulated against each of the independent variables and results are presented in Table 2. Overall, 22.2% of all the women reported having used a condom at their last sexual intercourse. However, enormous disparities in condom use existed by women’s characteristics. Of the women who reported that they were confident to negotiate condom use with their sexual partners, 27.9% reported that they actually used a condom at their last sexual encounter. This proportion was 6.9% among women who were not confident to negotiate condom use with their sexual partners and the difference was statistically significant (P<0.001). The level of condom use at the last sexual intercourse ranged from 12.4% among women currently married or living with partners as married to 54.9% among those who were single (P<0.001). In view of age, youngest women (<20 years) were the most condom users (48.0%) compared to the rest. The proportion using a condom declined sharply with increasing age as 23.8%, 15.5% and 11.2% among the 20-29, 30-39 and 40-49 year-olds respectively (P<0.001). In terms of religion, the extent of condom use was; 22.0% among Christian women, 23.4% among Muslim women, and 9.2% among followers of traditional and other beliefs (P=0.022). Condom use varied by household wealth status with women from rich households being the most condom users (25.6%). While this proportion was similar (24.8%) to that observed among women from socioeconomically middle households, only 17.4% of those who were from socioeconomically poor households actually used a condom at the last sexual intercourse (P<0.001). With regard to education, a highest proportion (52.3%) of condom users was found among those who had attained a secondary or higher education level. This proportion was 20.1% and 14.4% among women with primary education and those who had never been to school respectively (P<0.001).

Also, of the women who reported knowledge of reduced chances of contracting the HIV by using a condom every time people have sex, 24.1% actually used it at their last sexual intercourse. This proportion was 18.5% and 9.3% among women who did not agree with this claim and those who did not know about it respectively (P<0.001). Condom use was on the other hand affected by the number of sexual partners a woman had in the past 12 months. Near a half (47.6%) of the women who reported that they had multiple sexual partners in the past 12 months actually used a condom at their last sexual intercourse. The corresponding proportion among those who had no more than one partner was 20.0% (P<0.001). No association was found between condom use at the last sexual intercourse and district of residence (P=0.721).

### Reason for not using a condom

**Figure 1:**
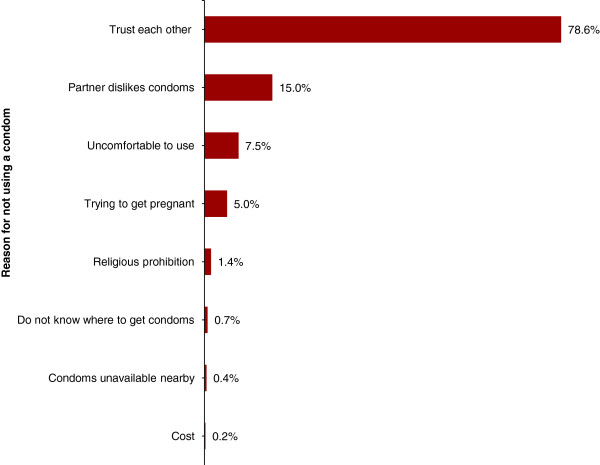
Reasons for not using a condom at the last sexual intercourse and the proportion of 15-49 year-old women reported each reason in three districts in Tanzania (n = 2,034), 2011.

During the survey, women who reported that they did not use a condom at the last sexual intercourse (n = 2,034) were subsequently asked to give reason(s) for that. Multiple responses of each woman were accommodatable, even though a very few women actually reported more than one reason (Figure 1). A majority of the women (78.6%) reported that they did not use a condom because they trust their sexual partners and vice versa. Other reasons reported for not using a condom at the last sexual intercourse and their corresponding proportions of women who reported each of them were: partner dislikes condoms (15.0%), condoms are uncomfortable to use (7.5%), trying to get pregnant (5%), religious prohibition (1.4%), not knowing where to get condoms (0.7%), condoms unavailable nearby (0.4%) and condoms are pricey (0.2%).

### Regression results of condom negotiation and condom use

Table 3 presents the effect of condom negotiation on condom use at the last sexual intercourse, controlled for marital status, age, education attainment, religion, household wealth status, knowledge of HIV prevention by condom use and number of sexual partners a woman had in the past 12 months preceding the survey. Our findings show that women who were confident to negotiate condom use with their sexual partners were significantly and independently 3.1 times more likely than women who were not confident to have used a condom at the last sexual intercourse (OR=3.13, 95% CI 2.22-4.41, P<0.001).

**Table 3:** **Mulitvariate logistic regression of the effect of condom negotiation on condom use at the last sexual intercourse among 15**-**49 year**-**old women in rural Tanzania**, **2011** (**n** = **2**,**557**)

	**Condom use at the last sexual intercourse**			
**Variable**	**Odds Ratio (OR)**	**Standard Error (SE)**	**95% Confidence interval (CI)**	**P - value**
**Negotiation of condom use**				
Not confident	1.00	--	--	--
Confident	3.13	0.55	2.22-4.41	<0.001
**Marital status**				
Currently married or living with a partner as married	1.00	--	--	--
Divorced/Widowed	3.64	0.65	2.56-5.17	<0.001
Single	4.09	0.58	3.10-5.40	<0.001
**Age group (in years)**				
<20	1.00	--	--	--
20-29	0.56	0.09	0.41-0.76	<0.001
30-39	0.49	0.08	0.34-0.68	<0.001
40-49	0.38	0.08	0.25-0.58	<0.001
**Educational attainment**				
Never been to school	1.00	--	--	--
Primary	0.96	0.15	0.70-1.31	0.780
Secondary and higher	2.04	0.45	1.31-3.15	0.001
**Religion**				
Christian	1.00	--	--	--
Muslim	1.07	0.12	0.86-1.33	0.561
Other (e.g. traditional etc.)	1.17	0.48	0.52-2.62	0.707
**Household wealth status**				
Rich	1.00	--	--	--
Middle	1.16	0.16	0.89-1.51	0.271
Poor	0.89	0.13	0.67-1.17	0.394
**Can people reduce their chances of contracting HIV by using a condom every time they have sex?**				
Yes	1.00	--	--	--
No	0.73	0.11	0.54-1.00	0.047
Don’t know	0.61	0.18	0.34-1.11	0.090
**Number of sexual partners a woman has had in the past 12 months**				
At most one	1.00	--	--	--
At least two (multiple partner)	3.32	0.58	2.36-4.68	<0.001

Goodness-of-fit test P = 0.259.

There were other independent correlates of condom use at the last sexual intercourse as follows: The odds of condom use at the last sexual intercourse among women ever married (currently divorced or widowed) was 3.6 times as high as that for women currently married (OR=3.64, 95% CI 2.56-5.17, P<0.001 ). Likewise, women who were single were 4.1 times more likely than currently married women to have used a condom at the last sexual intercourse (OR=4.09, 95% CI 3.10-5.40, P<0.001). In terms of age (in years), we observed a significant and sustained decline in the likelihood of condom use as age increased. With youngest (<20) women being a reference, the odds of condom use at the last sexual intercourse was 44% less likely among women aged 20-19 (OR=0.56, 95% CI 0.41-0.76, P<0.001), 51% less likely among women aged 30-39 (OR=0.49, 95% CI 0.34-0.68, P<0.001) and 62% less likely among women aged 40-49 (OR=0.38, 95% CI 0.25-0.58, P<0.001). Concerning education attainment, women who had at least a secondary education were twice as likely as women who had never been to school to have used a condom at the last sexual intercourse (OR=2.04, 95% CI 1.31-3.15, P=0.001). Further evidence of increased likelihood of condom use at the last sexual intercourse existed by multiple sexual partners, such that women who reported an experience of multiple sexual partners in the last 12 months were 3.3 times as likely as women who had not more than one partner in the same period to have used a condom at the last sexual intercourse (OR=3.32, 95% CI 2.36-4.68, P<0.001).

On the other hand, women who reported that people cannot reduce their chances of contracting HIV by using a condom every time they have sex were significantly 27% less likely compared to women who reported so to have used a condom at the last sexual intercourse (OR=0.73, 95% CI 0.54-1.00, P=0.047). However, women who reported that they do not know that people can reduce their chances of contracting HIV by using a condom every time they have sex were 39% less likely compared to women who agreed with the claim to have used a condom at the last sexual intercourse but there was no sufficient statistical evidence of this observation (OR=0.61, 95% CI 0.34-1.11, P=0.090). Finally, having controlled for other variables in the full model, condom use at the last sexual intercourse was not associated with religion and household wealth status.

## Discussion

This study examined whether a woman’s confidence or capability to negotiate condom use with her sexual partner(s) has any consequence on the actual use of condoms. The study also sought to describe the proportion of women who feel confident to negotiate condom use and prevalence of condom use at the last sexual intercourse in Tanzania. Findings reveal a large proportion of women being confident to negotiate condom use with their partners. Generally, condom use at the last sexual intercourse was significantly dependent on characteristics of the individual. A majority of the women who did not use condoms at the last sexual intercourse reported that they trust their partners.

Overall results show a strong evidence of increased likelihood of condom use by confidence to negotiate it. This observation remained adamantly significant even after controlling for marital status, age, education, religion, household wealth status, condom knowledge and number of sexual partners in the preceding 12 months. This suggests that a woman’s confidence or capability to speak for herself about condom use with her sexual partner represents her protection against acquisition of not only HIV/AIDS and other STIs but also unintended pregnancies. On the other hand, the results imply that women who lack confidence to negotiate condom use with their sexual partners may be exposed to unprotected intercourse, thus at risk of contracting STIs including HIV/AIDS and consequently being more vulnerable to adverse sexual and reproductive health outcomes. This is consistent with findings from other studies in which it is reported that a woman’s confidence to negotiate condom use correlates with higher levels of condom use [38,39]. The literature further shows that women who are in relationships where they have limited decision-making powers are less likely to use condoms than those with adequate control of their relationships [40,41].

Regarding marital status, condom use was lowest among women who were married or living with partners as married and highest among women who were single, and ever married. This observation persisted in the multivariate model, with both women who were single, and ever married being almost four times more likely than women who were married to have used a condom at the last sexual intercourse. This is consistent with extant evidence [42,43] showing that, in marriage, couples will use condoms most likely as a family planning method, not primarily for disease prevention purposes [44]. However, where one partner suspects or know that the other partner is not faithful or is infected with STIs especially HIV, condom use may be expected, although fear of breaking the relationship coupled with religious stance that condom use is a sin, makes it difficult for such married couples to ask their partners to use condoms [21]. One study in Malawi considered condom use in marriage as an ‘intruder’ in the domestic space [20], to mean that condom use in marriage threatens the relationships thus less expected for purposes other than pregnancy prevention. Condom use is denied due to claims that they connote mistrust or dearth of love intimacy [45,46]. Therefore, low condom use in marriage was expected, given the close association between marriage and fertility and the fact that condom use is not even one of the popular fertility control methods in Tanzania [27]. On the other hand, the increased condom use among unmarried and ever married women is likely due to perceived risk of contracting STIs including HIV and unintended pregnancies.

Age and condom use at the last sexual intercourse related inversely, with the likelihood of condom use declining rapidly and constantly with ageing and vice versa. This is consistent with the recent Tanzania Demographic and Health Survey findings [27]. The greater use of condoms observed among the youngest (<20) women is probably because of perceived risk of contracting STIs including HIV and unintended pregnancies because most of them were unmarried. Despite being low, condom use in subsequent age categories in which most women were married was probably for birth spacing purposes primarily, not as a disease control measure. This is also supported by the fact that over three-quarters of women who did not use condoms at the last sexual intercourse said that they did so because they trust their partners and 84.4% of them were married. Additionally, the likelihood of condom use in the oldest age category (≥40) was even much slim, a reflection of aging towards menopausal where fertility control mechanisms including condom use are rarely used because of declining reproductive capacity or fecundity with age [47].

Regarding education attainment, women with at least a secondary education were twice as likely as those who have never been to school to have used a condom at their last sexual intercourse. The likelihood of condom use was similar between women with primary education and those who have never been to school. The evidence of condom use with secondary or higher education is likely due to an imperative role that education plays on societal transformation and also that education enhances women’s self-esteem, self-confidence, ability to make decisions and freedom of expression [48] concerning their sexual and reproductive inclinations. This underscores a need to promote women’s education beyond primary school as a prerequisite for change (e.g. behavioural change) in all aspects of their life. Education functions as a one powerful input upon which numerous outcomes, including informed choices of safer sex options, upshot. Education is also acknowledged in the literature as a catalyst for change in gender relations [48,49].

Even though women who reported multiple sexual partners in the last 12 months were few, they were more likely than women who had not more than one sexual partner in the same period to have used a condom at the last sexual intercourse. Engaging in sexual relationships with multiple partners is a risky behaviour [27] and emphasis has always been centering on condom use at each risky intercourse to ensure protection against STIs including HIV [27,50]. Therefore, it may be because of perceived risk of contracting STIs especially HIV that women with multiple sexual partners were more likely to use condoms. This agrees with findings from another study in Tanzania, where high-risk sexual behaviour was associated with increased condom use [51].

Finally, condom use at the last sexual intercourse was less likely among women who reported that people cannot reduce their chances of contracting HIV by using a condom every time they have sex compared to those who did. This observation reflects a true context, since people may not use condoms unless they believe that condoms are capable of preventing transmission of HIV or pregnancy. This may be linked to condom misconceptions or negative outlook towards condom effectiveness [19] and condom use which some women, depending on their culture, values and norms, may be having. Therefore, it is important that interventions that promote condom use also highlight key issues about condoms effectiveness.

Unlike other HIV prevention methods such as male circumcision where a female partner benefits indirectly, condom use protects both partners. For uninfected women, it is the strategy that they have the most immediate ability to influence. A woman could ask her uninfected male partner to get circumcised in order to reduce his likelihood of acquiring HIV (from her, or from another partner); she could ask her already-HIV-infected partner to get on antiretrovirals (ARVs) or get on ARVs herself to lessen her chances of passing the infection on; and she could negotiate for condom use - condom use being the least technically and logistically demanding of the three, and also a strategy that can be accomplished within minutes.

### Limitations

Condom use was self-reported with no means to validate the responses other than probing the respondent. We understand that self-reports of sexual behaviour are often invalid and unreliable as already known [52-54]. Also these findings may not be generalized to the entire population of Tanzania since three districts only were studied. Similarly, no causal inferences may be drawn because temporality cannot be established in cross-sectional studies.

## Conclusions

Confidence to negotiate condom use is a significant predictor of actual condom use among women in rural Tanzania. This implies that women, especially unmarried ones, those in multiple partnerships or any women needing protection who are not confident to negotiate condom use may be open to an unprotected sexual intercourse thus at risk of contracting STIs including HIV/AIDS and unintended pregnancies. Therefore HIV/AIDS prevention programs in Tanzania should not only focus on increasing HIV transmission and prevention knowledge [55], but more importantly empower such women with condom negotiation skills. Given the incompatibility of condom use with marriage, faithfulness is necessary to reduce chances of contracting STIs and consequently reduce the need for condoms for disease prevention purposes within marriage.

## Competing interests

The authors declare that they have no competing interests.

## Authors' contributions

AE conceptualized the research question, designed the study, executed data analysis and wrote the manuscript drafts. AMK participated in designing the study, data analysis and critical review of the manuscript drafts. EJ, KR, AH and JFP designed the primary study and critically reviewed the drafts of the current manuscript together with HPM, JN, GS, KT and CB. All the authors read and approved the final draft of the manuscript.

## Pre-publication history

The pre-publication history for this paper can be accessed here:

http://www.biomedcentral.com/1471-2458/12/1097/prepub
